# Risk Factors Associated With Low Back Pain in Bangladesh: A Cross‐Sectional Study Conducted in 2023

**DOI:** 10.1002/hsr2.71151

**Published:** 2025-08-10

**Authors:** Mohammad Omar Faruk, Najma Begum, Kabir Hossain, Md. Rakib Rahman, Md. Sahidur Rahman, Sorif Hossain

**Affiliations:** ^1^ Department of Statistics Noakhali Science and Technology University Noakhali Bangladesh; ^2^ Institute for Intelligent Systems Research and Innovation Deakin University Waurn Ponds Australia; ^3^ One Health Center for Research and Action Chattogram Bangladesh; ^4^ Eastern Mediterranean Public Health Network (GHD|EMPHNET) Bangladesh Country Office Dhaka Bangladesh

**Keywords:** Bangladesh, disability, lifestyle‐related factors, low back pain (LBP), occupational factors, sociodemographic factors

## Abstract

**Background and Aims:**

Low back pain (LBP) is a chronic health condition that reduces quality of life and imposes a burden on individuals, societies, and governments. It is a leading cause of physical disabilities and musculoskeletal disorders. This study aimed to identify the risk factors for LBP and associated physical disabilities.

**Methods:**

This cross‐sectional study was conducted among 396 patients at the Center for the Rehabilitation of the Paralyzed (CRP) between April and August 2023. This study collected data on sociodemographic, occupational, and lifestyle‐related factors, along with the severity of LBP, which was rated using a Likert scale (0–10). The severity of pain was categorized into four groups based on disability levels: low disability with low intensity, low disability with high intensity, high disability with moderately limiting intensity, and high disability with severely limiting intensity. Descriptive analysis was conducted to summarize background characteristics, and *χ* ^2^ test used to examine the association between independent variables and LBP. Multinomial logistic regression used to identify significant risk factors for LBP. All analyses were performed using Statistical Package for the Social Sciences (SPSS), Version 25.0 (IBM).

**Results:**

The results showed that factors such as using soft pillows, physical inactivity, using soft leather chairs, and having a job that involves prolonged standing increased the risk of LBP and related disability. Conversely, factors that decrease the risk include younger age, lower education level, being unmarried, being a student, not having diabetes, no history of discomfort while sitting or trauma, absence of leisure time activities (hanging with friends), no job‐related stress, and wearing light or medium‐weight shoes.

**Conclusion:**

The study found a high prevalence of LBP among participants, and suggested the importance of implementing the necessary health precautions to address the risk factors that are associated with it.

## 1 Introduction

Low back pain (LBP) is a global public health concern, affecting ~619 million people in 2020, with projections reaching 843 million by 2050 [1]. It is characterized by pain, muscle tension, or stiffness between the low ribs and buttock, can be as acute (< 6 weeks), sub‐acute (6–12 weeks), or chronic (> 12 weeks) [1, 2, 3]. LBP has significant physical and psychological consequences, being a leading cause of disability [4] and contributing to physical disabilities in 11%–12% of individuals [2, 3, 5]. According to an estimation, the number of disabilities caused by LBP increased by 54% between 1990 and 2015 [6, 7]. As the most common musculoskeletal disorder globally, LBP affects individuals of all ages, including adolescents and young adults [1]. Beyond physical conditions, negative self‐perception, fear‐avoidance beliefs, social stress, catastrophic thinking, depression, anxiety, and suicidal thoughts and attempts are highly evident among patients with chronic LBP [2, 7, 8, 9]. Chronic LBP further diminishes mental well‐being, reduces life expectancy, and negatively impacts physical health‐related quality of life [2, 10]. It is not only a health issue but also a social and economic burden for populations of all ages [3].

In low‐ and middle‐income countries (LMICs), LBP remaining underrecognized despite its substantial burden [7, 11]. In Bangladesh, the prevalence of LBP was reported at 6.6% in rural areas, 9.9% in urban areas, and 9.2% in affluent urban areas [12]. Additionally, Bangladeshi individuals face a higher risk of disability due to LBP, because of the involvement of heavy weight lifting in manual labor, particularly rickshaw pulling, day laborers, and housemaids [3, 7].

Recent research has shown that spinal pain has become a major occupational health issue, particularly for physiotherapists and other healthcare professionals. For example, a study of physiotherapists in Poland revealed that 91.7% of participants had spinal pain, with the most common areas being the lumbosacral and cervical regions. The study evaluated the degree of disability brought on by neck and lower back pain using validated instruments like the Oswestry Disability Index (ODI) and the Neck Disability Index (NDI). Additionally, it demonstrated that physical activity significantly reduced the frequency of pain episodes, while poor ergonomics, prolonged standing, lifting, and bending were major contributing factors [13]. The necessity of the current study to investigate the risk factors of LBP and related disability in the Bangladeshi context is supported by these findings, which highlight the significance of assessing lifestyle and occupational factors in the development of spinal pain.

Despite earlier research on LBP, few studies have thoroughly examined socioeconomic, occupational, and lifestyle‐related risk factors in Bangladesh. The objectives of this study are to fill this gap by investigating these factors and their impact on the prevalence of LBP. This study provides evidence‐based insights into the prevalence and risk factors of LBP, allowing policymakers to develop customized healthcare policies and address the public health problems caused by LBP.

## 2 Methods

### 2.1 Area and Population

This cross‐sectional study was conducted among patients receiving treatment at the Center for the Rehabilitation of the Paralyzed (CRP) in Savar, Mirpur, Chattogram, and Mymensingh from April 10, 2023 to August 25, 2023. Personal communication was used to gather data because it allowed for direct contact with patients and a deeper understanding of the conditions, treatment experiences, and related factors of the patients.

### 2.2 Inclusion Criteria

Patients receiving physiotherapy or treatment at CRP who had musculoskeletal problems (e.g., shoulder stiffness, arthritis, or back pain) were included. Patients with postoperative recovery conditions unrelated to LBP, severe neurological disorders, or incomplete medical records were not included.

### 2.3 Sample Size Determination

The required sample size for this study was determined using Cochran's formula [14], with a 5% margin of error:

$$n=\frac{z^{2}p(1-P)}{d^{2}}=\frac{{\left(1.96\right)}^{2}\times 0.5\times (1-0.5)}{{\left(0.05\right)}^{2}}=384.16\approx 384$$

where *n* = required sample size, *Z* = 1.96 for a 95% confidence interval, *d* = 0.05 (margin of error), and *p* = 0.5 (prevalence estimate as no prior study found).

The minimum sample size was 384. However, after eliminating the incompletes, 396 data were selected for further analysis. The proportion of subjects in different centers was determined by patient flow, geographical coverage, and accessibility to ensure a diverse and representative sample. Personal communication helped include community‐based patients, providing deeper insights into their treatment experiences. The samples were distributed as follows: 117 patients from Savar, 79 from Mirpur, 64 from Chattogram, 27 from Mymensingh, and 109 through personal communication. Personal communications provide a diverse representation, particularly from patients who are referred for community or home‐based treatment.

### 2.4 Sampling and Data Collection Procedures

A convenient nonprobability sampling technique was used to collect information for this study. Information was gathered from the respondents using a well‐structured questionnaire. Face‐to‐face interviews were used to collect data using a structured questionnaire that was created after expert consultation and a review of the literature. The questionnaire was translated into Bangla to make it easily understandable to participants whose mother tongue was Bangla. In order to reduce bias, all interviewers received standardized training on how to collect data. A pilot study was conducted to check the validity and consistency of the questionnaire and included 30 patients who were excluded from the final survey. Based on the pilot survey, some questions were excluded, some were modified, and 53 were selected. The questionnaire was categorized into four sections: sociodemographic factors, lifestyle‐related factors, work‐related factors, and pain assessment. The pain assessment section used a Likert scale (0–10), where 0 represented *no pain* and 10 represented *extreme pain*. An open‐ended question was included at the end to allow participants to report the likely cause of their lower back pain. A total of 25 skilled interviewers participated in the study to collect data. These interviewers are students from the Department of Statistics at Noakhali Science and Technology University and received comprehensive training on standard operating procedures from the research author. They were assigned to various locations, including Savar, Mirpur, Chattogram, and Mymensingh, and handled personal communication cases with five interviewers at each center. Standardized training was implemented to ensure data collection consistency and minimize bias.

### 2.5 Outcome Variable

**Figure 1:**
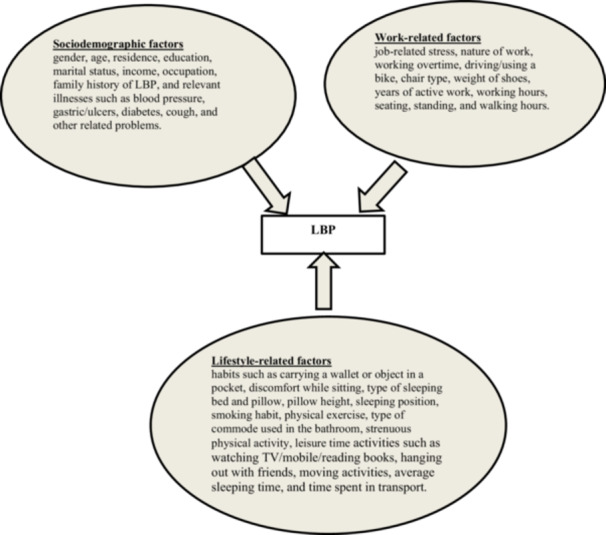
Conceptual framework of risk factors influencing LBP.

LBP was the outcome variable of this study. The LBP was rated through the Likert scale (0–10), in which the numerical numbers “0” to “10” represent the level of pain, from *no pain* to *extreme pain*. This variable was categorized into four hierarchical classes based on pain [15]. Descriptions of these categories are provided in Table 1. A conceptual framework of LBP and its associated factors is illustrated in Figure 1.

**Table 1:** The pain intensity score and pain characteristics of pain categories (grades).

Category	Pain characteristics	Description
Grade I	Low disability‐low intensity	Pain intensity score < 50
Grade II	Low disability‐high intensity	Pain intensity score ≥ 50
Grade III	High disability‐moderately limiting	3–4 disability points
Grade IV	High disability‐severely limiting	5–6 disability points

### 2.6 Independent Variables

This study considered three categories of independent variables: sociodemographic, lifestyle‐related, and work‐related factors. Sociodemographic variables included sex (male, female), age (18–30, 31–40, 41–50, 51–60, 60+), place of residence (rural, urban), education (no formal education, primary, secondary, higher secondary, graduate, postgraduate), marital status (married, unmarried, divorced/widowed/separated), income in thousand (0, 1–10, 11–25, 26–40, 40+), occupation (service holder, housewife, teacher, business, student, others) [16, 17], family history of LBP (parents, sibling, other) [18, 19], and relevant illnesses such as blood pressure (yes, no), gastric/ulcers (yes, no), diabetes (yes, no), cough (yes, no), and other related problems (yes, no) [20, 21].

Lifestyle‐related factors included habits such as carrying a wallet or object in a pocket (back pocket, front pocket, none), discomfort while sitting (yes, no), type of sleeping bed (soft, moderate, hard) and type of pillow (soft, moderate, hard), pillow height (low, moderate, high) [22], sleeping position (left/right, straight, back) [23], physical exercise (none, occasionally, frequent, regular), type of commode used in the bathroom (low, high, others), strenuous physical activity (not at all, rarely, sometimes, regular), leisure time activities such as watching TV/mobile/reading books, hanging out with friends, moving activities, average sleeping time (4.00, 5.00, 6.00, 6.50, 7.00, 8.00, 9.00), time spent in transport (0–1, 1–2, 2+) and smoking habit (none, occasionally, frequent, regular) [24].

Work‐related factors included job‐related stress (never, rarely, frequently, always), type of chair used in the workplace (plastic chair, soft leather/cushion chair, wooden chair, no use of chair), nature of work (standing, sitting, frequently movement), weight of shoe in last 6 months (light, medium, heavy), bike drive/usage (none, occasionally, frequent, regular), overtime work (never, sometimes, regularly), total number of years actively worked (0–10, 10–20, 20–30, 30–40, 40+), daily seating hours (0–4, 4–8, 8–12), working hours (1–4, 5–8, 9–12), daily standing hours (0–3, 3–6, 6–9), and daily walking hours (0–1, 1–2, 3).

Job‐related stress is the mental or emotional strain resulting from work demands, workload, and their work environment. It is commonly measured using the Job Stress Scale, where participants rate their stress levels as never stressed, rarely, frequently, and always stressed. This assessment helps identify the key factors contributing to stress in the workplace [11]. Nature of work such as standing, sitting, and frequent movement can contribute to the development of LBP by causing repetitive strain or long‐term static postures [25]. To evaluate the effect of daily work duration on LBP, participants were divided into three groups: 1–4, 5–8, and 9–12 h. Additionally, Individuals were classified as 0–10, 10–20, 20–30, 30–40, and 40+ years based on their active work duration [26].

### 2.7 Ethics Approval and Consent to Participate

Ethical approval was obtained from the Ethical Review Committee of Noakhali Science and Technology University, Bangladesh. The CRP authorized conducting the study. All participants gave their informed consent prior to enrolling in the study, ensuring that they were aware of its nature and goal.

### 2.8 Statistical Analysis

Data were carefully checked for completeness and consistency before being cleaned, coded, and entered for statistical analysis. Descriptive statistics were carried out by presenting the sociodemographic, lifestyle‐related, and work‐related factors as frequencies and percentages. The *χ* ^2^ test was used to identify the association between these factors and LBP. A multinomial logistic regression was applied to assess the influence of the independent variable on LBP. A two‐sided *p* < 0.05 was considered statistically significant. Data were analyzed using Statistical Package for the Social Sciences (SPSS), Version 25.0 (IBM) [27].

## 3 Statistical Test

### 3.1 Chi Square Test

The *χ* ^2^ test determines whether two categorical variables are significantly correlated. In this study, we examine the association between the categorical variables and LBP using *χ* ^2^ test of independence.

H_0_: There is no association between the variables.

H_1_: There is a significant association between the variables.

We conduct the test of hypothesis at 5% level of significance.

Test statistic: ${ꭓ}^{2}=\sum \frac{{\left(O-E\right)}^{2}}{E}$

where *O *= observed frequency and *E *= expected frequency.

If *p* value < level of significance (0.05), then we reject the H_0_, otherwise accept H_0_.

### 3.2 Multinomial Logistic Regression

Multinomial logistic regression is employed when there are more than two categorical outcomes for the dependent variable. It uses predictor variables to model the likelihood of each category occurring.

Let us consider the following hypothesis,

Null hypothesis (H_0_): There is no significant impact of the predictor variables on the categorical outcome.

Alternative hypothesis (H_1_): There is a significant impact of the predictor variables on the categorical outcome.

We conduct the test of hypothesis at 5% level of significance.

The likelihood of category *j* (in relation to a reference category) for a dependent variable *Y* with *K* categories is represented as follows:

$$\log(\frac{P(Y=j)}{P(Y=\mathrm{reference})}=\beta_{0}+\beta_{1}X_{1}+\beta_{2}X_{2}+\ldots +\beta_{n}X_{n}$$

where P(Y=j)= probability of *j* category, P(Y=reference)= probability of reference category, $\beta_{0}=$ intercept, $\beta_{1},\;\beta_{2},\;\beta_{n}=$ regression coefficients for $X_{1},\;X_{2},\;\mathrm{and}\;X_{n}$.

If *p* value < level of significance (0.05), then we reject the H_0_, otherwise accept H_0_.

## 4 Result

### 4.1 Prevalence of Low Back Pain

**Figure 2:**
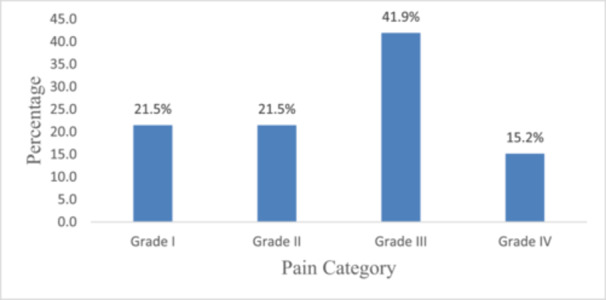
Prevalence of pain across different grades.

Figure 2 shows the prevalence of LBP by grades. The majority of participants (41.9%) reported Grade III pain, which is defined as having moderate limitations and high disability, while 15.2% reported Grade IV pain, which is defined as having severe limitations and high disability. Both Grade I and Grade II pain, which are linked to low disability but differing degrees of intensity, were reported by 21.5% of participants.

### 4.2 Sociodemographic Characteristics

A total of 396 individuals participated in this survey. Table 2 presents the participants' sociodemographic characteristics. Of the total respondents, 218 (55.05%) were female and 178 (44.9%) were male. In total, 95 (24.0%) participants belonged to the age group of 41–50 years and lived in urban areas, which accounted for 270 (68.2%) of the total respondents. Nearly three‐fourths (74.5%) of the participants were married, and 22.2% had secondary education. In terms of occupation, 127 (32.1%) participants were housewives. However, the family members (others) of the respondents frequently reported LBP, accounting for 366 (92.4%) cases.

**Table 2:** Sociodemographic characteristics of the study participants.

Variables	Categories	Frequency	Percentage
Gender	Female	218	55.1
	Male	178	44.9
Age of the participants	18–30	89	22.5
	31–40	88	22.2
	41–50	95	24.0
	51–60	78	19.7
	60+	46	11.6
Place of residence	Rural	126	31.8
	Urban	270	68.2
Education	No formal education	49	12.4
	Primary	64	16.2
	Secondary	88	22.2
	Higher secondary	76	19.2
	Graduate	81	20.5
	Postgraduate	38	9.6
Marital status	Married	295	74.5
	Unmarried	63	15.9
	Divorced/widowed/separated	38	9.6
Income	0	117	29.5
	1–10	43	10.9
	11–25	81	20.5
	26–40	90	22.7
	40+	65	16.4
Occupation	Service holder	100	25.3
	Housewife	127	32.1
	Teacher	30	7.6
	Business	33	8.3
	Student	41	10.4
	Others	65	16.4
Family history (parents) of LBP	No	256	64.6
	Yes	140	35.4
Family history (siblings) of LBP	No	330	83.3
	Yes	66	16.7
Family history (others) of LBP	No	366	92.4
	Yes	30	7.6
Had other relevant illness (blood pressure)	No	256	64.6
	Yes	140	35.4
Had other relevant illness (gastritis/ulcer)	No	270	68.2
	Yes	126	31.8
Had other relevant illness (diabetes)	No	295	74.5
	Yes	101	25.5
Had other relevant illness (cough)	No	344	86.9
	Yes	52	13.1
Had other's problem	No	364	91.9
	Yes	32	8.1

### 4.3 Lifestyle‐Related Characteristics

Table 3 presents lifestyle‐related characteristics of the study participants. More than 63.9% of the respondents did not keep anything in their front or back pockets. Approximately 45.2% of participants experienced discomfort during seating or trauma. Most respondents (63.1%) used a low commode in their bathrooms. A significant number of respondents used moderately softened beds (62.6%) and pillows of moderate height (67.7%) to sleep on, and the majority of them slept in either the right or left position (67.4%). Most participants (73.0%) did not smoke, and 36.9% did not engage in physical exercise.

**Table 3:** Lifestyle‐related characteristics of the study participants.

Variables	Categories	Frequency	Percentage
Keeping a wallet or something in the pocket	Back pocket	108	27.3
	Front pocket	35	8.8
	None	253	63.9
Have you ever had an incidence of discomfort sitting or trauma?	Yes	179	45.2
	No	164	41.4
	Don't know	53	13.4
Type of sleeping bed	Soft	93	23.5
	Moderate	248	62.6
	Hard	55	13.9
Pillow height	Low	86	21.7
	Moderate	268	67.7
	High	42	10.6
Type of pillow	Soft	130	32.8
	Moderate	243	61.4
	Hard	23	5.8
Sleeping position	Left/right	267	67.4
	Straight	104	26.3
	Back	25	6.3
Smoking habit	None	289	73.0
	Occasionally	21	5.3
	Frequent	26	6.6
	Regular	60	15.2
Physical exercise	None	146	36.9
	Occasionally	64	16.2
	Frequent	69	17.4
	Regular	117	29.5
Type of exercise	No exercise	160	40.4
	Light	165	41.7
	Medium	65	16.4
	Heavy	6	1.5
Improper weight lifting	None	195	49.2
	Occasionally	114	28.8
	Frequent	78	19.7
	Regular	9	2.3
Commode type used in bathroom	Low	250	63.1
	High	142	35.9
	Others	4	1.0
Strenuous physical activity	Not at all	182	46.0
	Rarely	102	25.8
	Sometimes	96	24.2
	Regular	16	4.0
Leisure time activity (watching TV/mobile/reading books)	No	80	20.2
	Yes	316	79.8
Leisure time activity (hanging with friends)	No	314	79.3
	Yes	82	20.7
Leisure time activity (moving activities)	No	377	95.2
	Yes	19	4.8
Average sleeping time	4.00	32	8.1
	5.00	38	9.6
	6.00	131	33.1
	6.50	3	0.8
	7.00	93	23.5
	8.00	70	17.7
	9.00	29	7.3
Time spent in transport (in hours)	0–1	329	83.1
	1–2	33	8.3
	2+	34	8.6

### 4.4 Work‐Related Characteristics

As shown in Tables 4, 33.3% of the participants experienced frequent stress in their jobs due to various work‐related characteristics. Additionally, the majority of respondents (51.3%) frequently moved to jobs. In terms of seating arrangements, 26.8% of the participants used wooden chairs and 50.3% wore medium‐weight shoes daily. Almost half of the participants (45.7%) sat for 4–8 h daily, while more than half of them (63.4%) stood up to 3 h daily.

**Table 4:** Work‐related characteristics of the study participants.

Variables	Categories	Frequency	Percentage
Job‐related stress	Never	91	23.0
	Rarely	109	27.5
	Frequently	132	33.3
	Always	64	16.2
Type of chair used in the workplace	Plastic chair	96	24.2
	Soft leather/cushion chair	93	23.5
	Wooden chair	106	26.8
	No use of chair	101	25.5
Nature of work	Seating	133	33.6
	Standing	60	15.2
	Frequent movement	203	51.3
Weight of shoe in last 6 months	Light	171	43.2
	Medium	199	50.3
	Heavy	26	6.6
Bike drive/usage	None	294	74.2
	Occasionally	35	8.8
	Frequent	29	7.3
	Regular	38	9.6
Overtime work	Never	211	53.3
	Sometimes	158	39.9
	Regularly	27	6.8
Total number of years actively worked	0–10	248	62.6
	10–20	67	16.9
	20–30	58	14.6
	30–40	19	4.8
	40+	4	1.0
Daily seating hours	0–4	177	44.7
	4–8	181	45.7
	8–12	38	9.6
Working hours	1–4	66	16.7
	5–8	261	65.9
	9–12	69	17.4
Daily standing hours	0–3	251	63.4
	3–6	104	26.3
	6–9	41	10.4
Daily walking hours	0–1	236	59.6
	1–2	84	21.2
	3	76	19.2

### 4.5 Factors Associated with LBP

**Figure 3:**
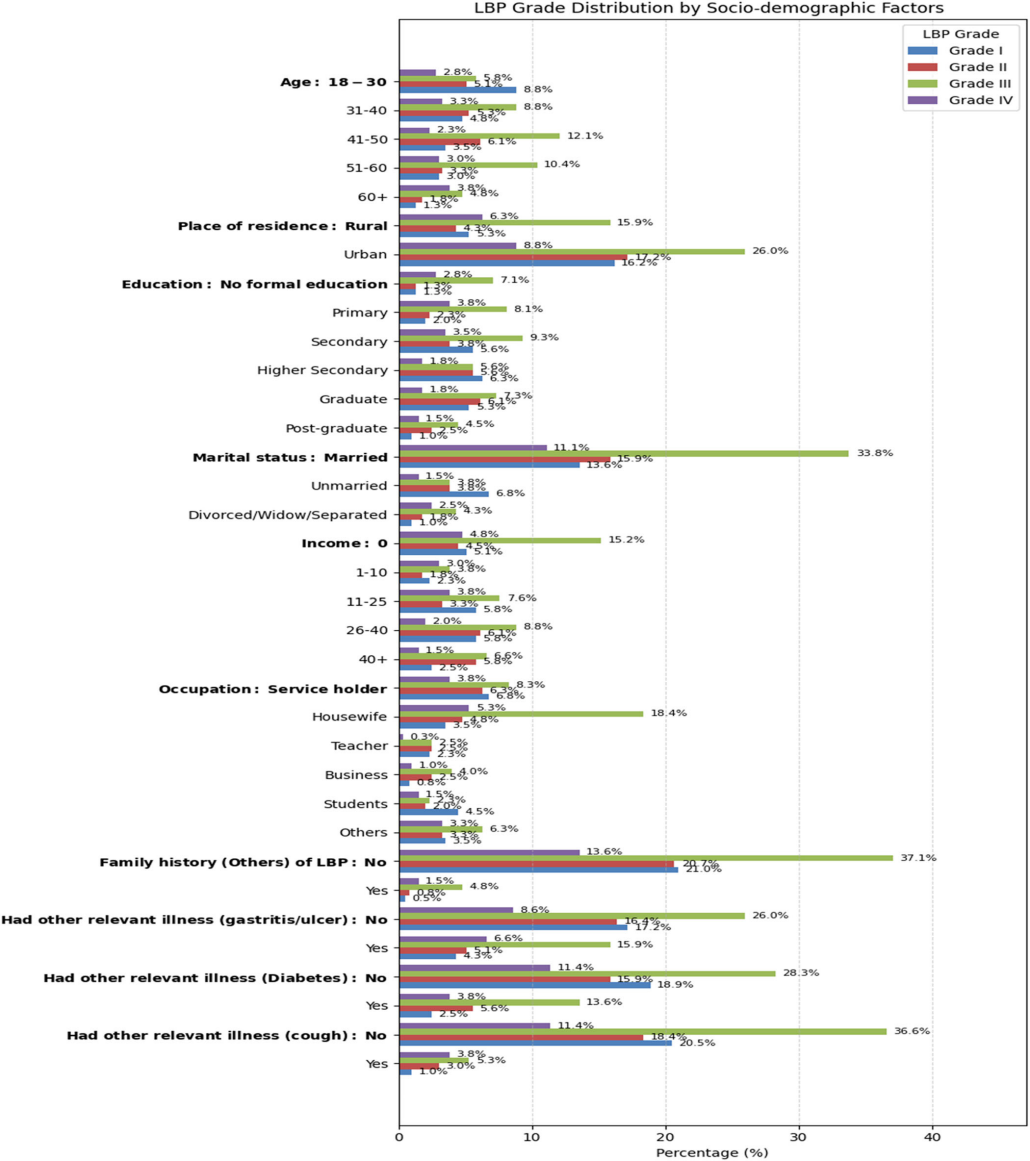
Distribution of LBP grades across various sociodemographic factors.

**Figure 4:**
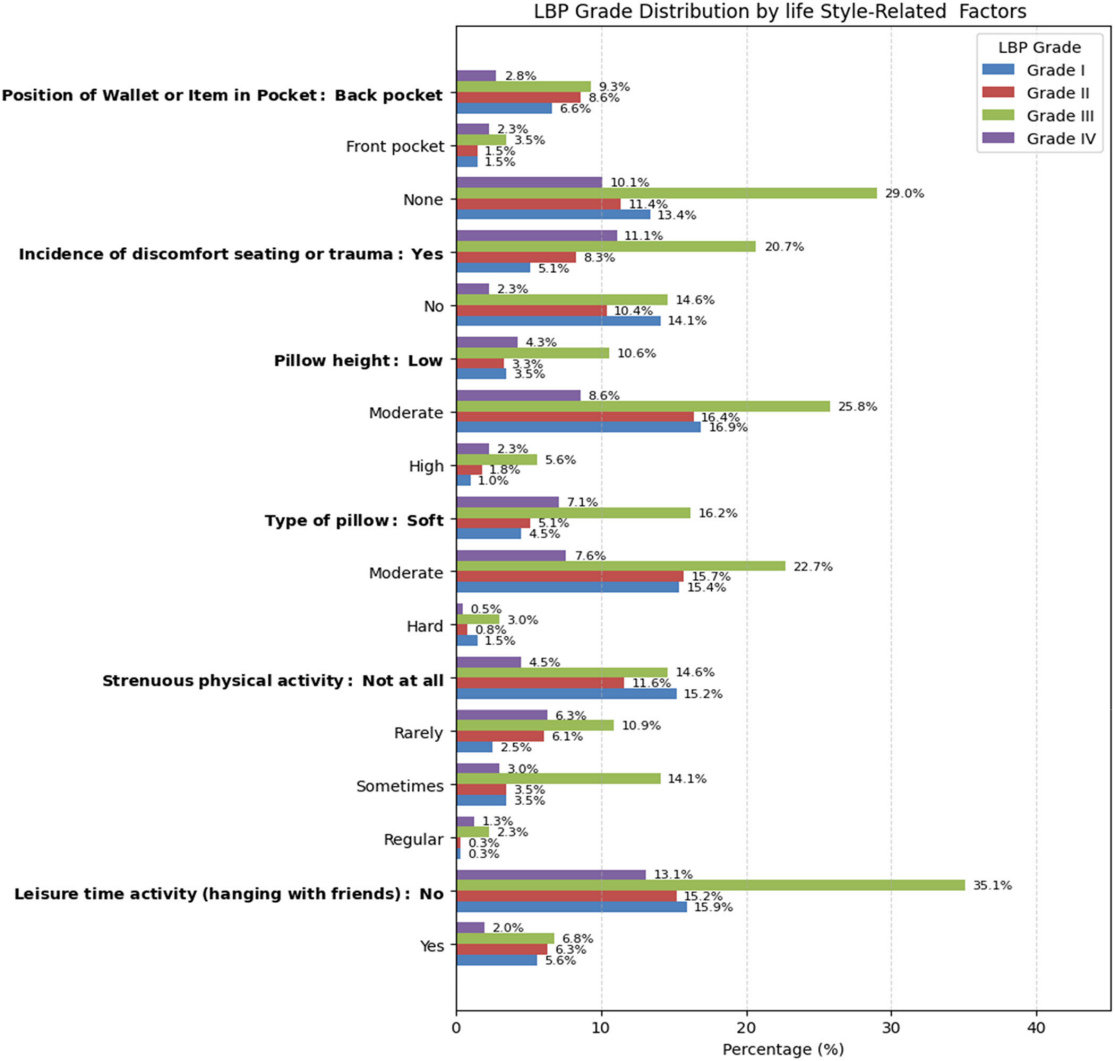
Distribution of LBP grades across various lifestyle‐related factors.

**Figure 5:**
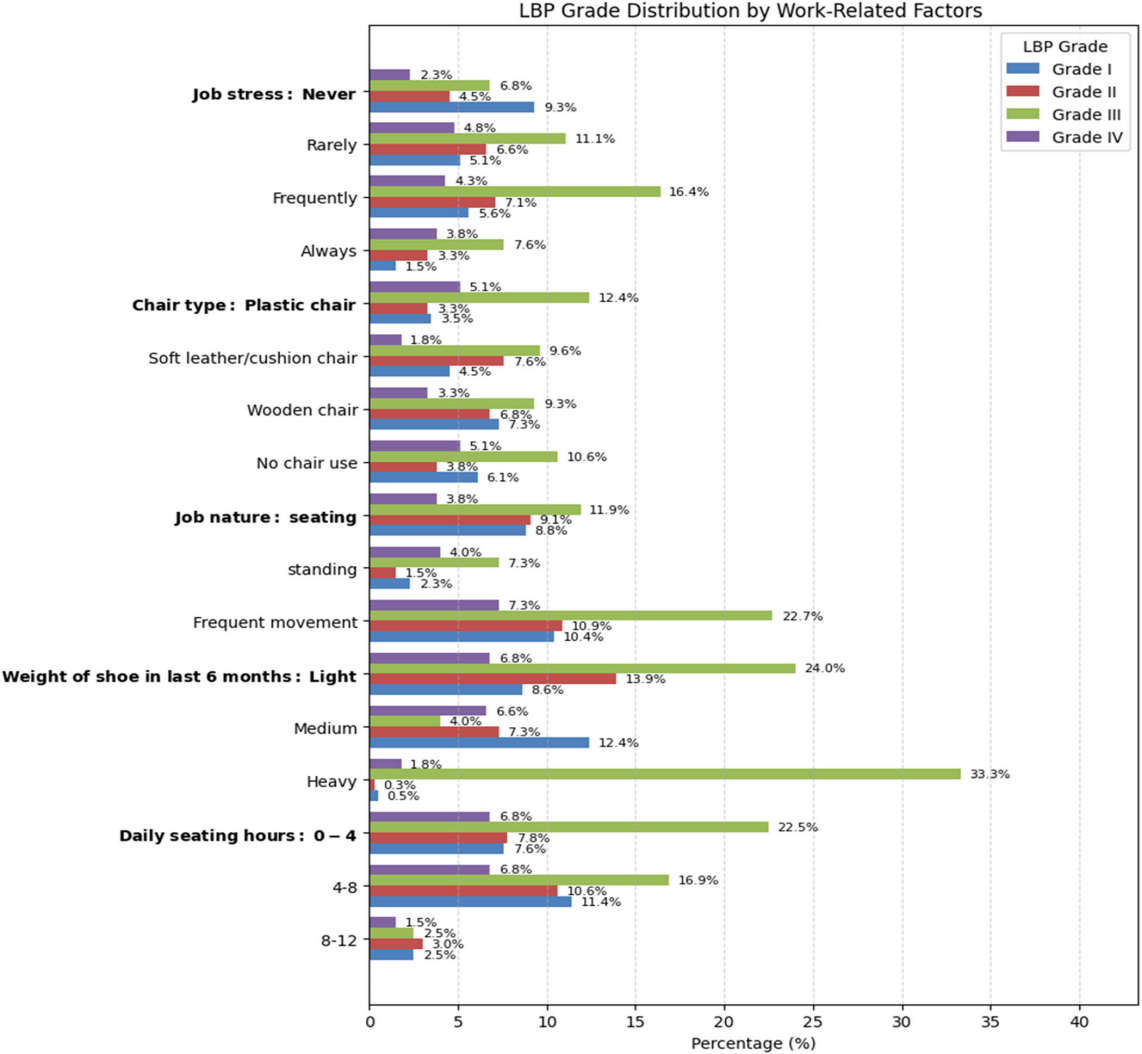
Distribution of LBP grades across various work‐related factors.

The bivariate analysis indicates that several factors were associated with LBP. The results are visually presented in Figures 3, 4, 5, and the complete statistical outputs are provided in Table S5.

The association between socioeconomic factors and LBP is presented in Figure 3. Based on the result, LBP was significantly associated with age (*p* < 0.001), place of residence (*p* = 0.005), marital status (*p* < 0.001), educational status (*p* < 0.001), employment status (*p* < 0.001), income (*p* = 0.005), family history of LBP among the others (*p* = 0.025), and relevant illnesses such as gastritis/ulcer, diabetes, and cough (*p* < 0.01).

Similarly, Figure 4 shows the association between lifestyle‐related factors and LBP. Keeping a wallet or other items in the pocket (*p* = 0.026), discomfort while sitting or due to trauma (*p* < 0.001), pillow height and type (*p* = 0.021 and 0.004), strenuous physical activity (*p* < 0.001), and leisure time spent hanging out with friends (*p* = 0.026) were statistically significant predictors of LBP.

Figure 5 illustrates the association between job‐related factors and LBP. According to the result, LBP was also associated with job‐related stress (*p* < 0.001). Additionally, the chair type used in the working area (*p* = 0.002) was significantly associated with LBP. Furthermore, the nature of the job (*p* = 0.008), weight of the shoes (*p* < 0.001), and daily sitting hours (*p* = 0.049) were strongly related to LBP.

### 4.6 Determinants of Low Back Pain

**Figure 6:**
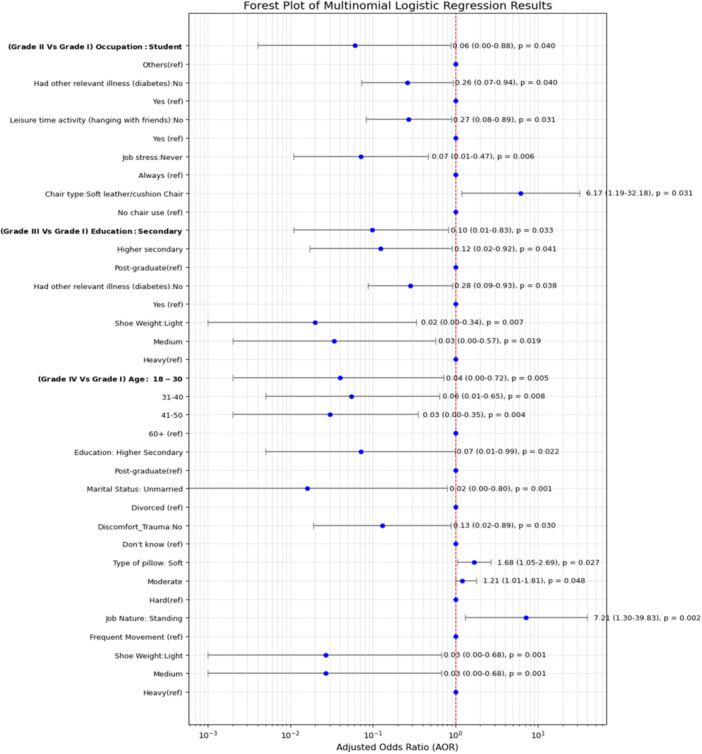
Forest plot of multinomial logistic regression results.

The results of multinomial logistic regression analysis showed that several factors significantly predicted the occurrence of LBP, as illustrated in Figure 6, with full model estimates provided in Table S6. These included age, educational level, marital status, employment status, relevant illnesses such as diabetes, discomfort while sitting or due to trauma, type of pillow used, strenuous physical activity, leisure time spent hanging out with friends, job‐related stress, type of chair used in the working area, nature of the job, and weight of shoes.

According to the analysis, participants aged 18–30, 31–40, and 41–50 had a lower risk of high disability, severely limiting LBP by 96% (AOR = 0.040, 95% CI: 0.002–0.724, *p* = 0.030), 95% (AOR = 0.055, 95% CI: 0.005–0.647, *p* = 0.022), and 97% (AOR = 0.030, 95% CI: 0.002–0.353, *p* = 0.006), respectively, compared to individuals aged over 60 years. Furthermore, participants with secondary (AOR = 0.098, 95% CI: 0.011–0.831, *p* = 0.033) and higher secondary education (AOR = 0.123, 95% CI: 0.017–0.916, *p* = 0.040) had a lower likelihood of having high disability with moderate back pain, and those with higher secondary education also had a 93% lower chance of being highly disabled with severe limitation of back pain than those who attained postgraduation. Additionally, unmarried respondents were 98% less likely to have a high disability with severe back pain (AOR = 0.016, 95% CI: 0.000–0.796, *p* = 0.037) than divorced/widowed/separated respondents, and people who had an occupation as students decreased the odds of low disability with high‐intensity back pain by 94% (AOR = 0.061, 95% CI: 0.004–0.881, *p* = 0.040) compared to those who had other occupations. The study also found that diabetes is a significant factor in LBP, with individuals without diabetes having a 0.262 times lower risk (AOR = 0.262, 95% CI: 0.075–0.919, *p* = 0.037) of having a low disability with high intensity and 0.284 times lower risk (AOR = 0.284, 95% CI: 0.084–0.958, *p* = 0.042) of having a high disability with moderate back pain.

The use of soft pillows was associated with 68% (AOR = 1.684, 95% CI: 1.055–2.688, *p* = 0.027) and 21% (AOR = 1.212, 95% CI: 1.014–1.806, *p* = 0.048) higher likelihood of having a high disability with severe back pain compared to those who use hard pillows. Participants who were rarely physically active were 51 times more likely (AOR = 51.113, 95% CI: 2.218–1177.928, *p* = 0.014) to have low disability with high‐intensity back pain than those who were regularly physically active. On the other hand, 73% (AOR = 0.272, 95% CI: 0.083–0.890, *p* = 0.032) had a lower risk of having a low disability with high‐intensity back pain than those with leisure time activities.

Participants who had never experienced stress in their jobs had a 0.072 times lower risk (AOR = 0.072, 95% CI: 0.008–0.650, *p* = 0.019) of low disability with high‐intensity back pain than those who always felt stress. Using soft leather chairs in the working area increased the likelihood of having a low disability with high‐intensity back pain by about 6.174 times (AOR: 6.174, 95% CI: 1.185–32.187) compared to those who do not use chairs. People whose working nature involves standing are 6.952 times more likely (AOR = 6.952, 95% CI: 1.083–44.638, *p* = 0.041) to have a high disability with severe back pain than those who frequently move in their work. Finally, the odds of LBP were lower among individuals who used light and medium‐weight shoes, with high disability, moderate back pain, and high disability with severe back pain, compared to those who used heavy shoes.

## 5 Discussion

The aims of this study were to investigate factors associated with LBP and pain‐related disability in Bangladesh. The result of this study indicates that age is a significant factor associated with severe LBP and severe disability. Research suggests that individuals aged 60 years or older are more susceptible to severe LBP, whereas younger age groups have a lower likelihood of experiencing it. This finding is supported by multiple studies conducted in various countries, including Ethiopia, China, Ghana, India, Mexico, South Africa, the Russian Federation, Tunisia, and England [28, 29, 30, 31, 32]. One study found that nurses over the age of 31 were twice as likely to experience LBP than their younger counterparts [28]. Similarly, another study found that advanced‐aged individuals had the highest tendency to experience LBP [29]. These findings are consistent with a study conducted by Thomas and colleagues, who investigated musculoskeletal conditions [30].

In this study, unmarried individuals were found to have a low risk of experiencing high disability with severe back pain than those who are widowed or divorced. This result is consistent with a previous population survey study that found widowed or divorced respondents were more prone to having LBP in both sexes [33]. In a similar vein, research on the Iranian population has revealed that married people are more likely than single people to experience LBP [34].

Education was identified as a significant risk factor. Individuals with a higher level of education are less likely to experience high disability with moderate and severe back pain than those who are illiterate. This finding is in line with those of previous studies [32, 35, 36]. An earlier study conducted in the United States claimed that seniors with less education had worse physical disability and severe back pain, while another study found that those suffering from chronic back pain were more likely not to have a college degree. The analysis showed that individuals without diabetes had a low likelihood of experiencing high disability and moderate back pain. This finding is consistent with a critical literature review that found that diabetes increases the risk of LBP [37]. Additionally, the survey found that regular physical activity could be a protective factor against LBP. This finding is also supported by previous studies showing that regular weekly physical activity can reduce the risk of LBP [6, 38]. According to the American Physical Therapy Association guidelines, moderate‐ to high‐intensity exercises are recommended for LBP without pain, whereas low‐intensity exercises are recommended for those with generalized pain [39]. The investigation revealed that students are less likely to develop low disability and low‐intensity pain than those in other occupations. This finding is similar to previous studies conducted in Malaysia [40], Bangladesh [41], and India [42], where 60% of hotel workers, 62% of industrial workers, and 60% of industrial workers, respectively, reported LBP prevalence.

People who work in a standing position are more likely to suffer from high disability and severe limiting LBP than those who move frequently during work. A previous study conducted in China found that standing for long periods at work was a risk factor for LBP [6]. The study also showed that participants who never felt stressed in their jobs had a low risk of experiencing low disability with high‐intensity back pain compared to those who felt stressed frequently. This finding is consistent with previous studies that have associated psychological stress with LBP in various working populations [43]. Using soft leather chairs in the workplace for sitting increases the likelihood of experiencing low disability with high‐intensity pain compared with those who do not use chairs. In general, previous studies with contrasting results on sitting time at work were not found to be associated with LBP [44]. However, a few studies with similar results have examined the association between total sitting time and LBP [45]. This study suggests that physical activity during leisure time reduces the risk of low disability with high‐intensity back pain. A Norwegian prospective study also revealed a similar association between physical activity during leisure time and the risk of chronic LBP [46].

Our findings suggest that individuals who wear light‐ and medium‐weight shoes are at a low risk of experiencing high disability due to moderate or severe back pain than those who wear heavy shoes. A study showed that wearing unstable shoes helped nurses with LBP and disability, and they may be useful in the process of recovering from back pain. [47, 48].

## 6 Conclusion

In Bangladesh, LBP is a common problem that impacts individuals across all ages. This cross‐sectional study found several variables that were significantly associated with LBP and related disability including age, education level, marital and employment status, diabetes, discomfort while sitting or due to trauma, use of soft pillows and leather chairs, physical inactivity, lack of leisure time activities, job‐related stress, and wearing heavy shoes.

However, this study has limitations, such as its reliance on self‐reported data, which may introduce recall bias since participants might not accurately remember every detail. Additionally, the study lacked clinical tests to validate the intensity of LBP. Furthermore, the cross‐sectional design prevents demonstrating causal links between LBP and risk factors. Despite these drawbacks, the study offers insightful information about risk factors of LBP in Bangladesh, which can guide targeted interventions and public health initiatives. According to the findings, specific awareness‐raising and intervention techniques emphasizing stress management, ergonomic workplace procedures, and physical activity promotion may help lower the risk of LBP and related disabilities. Future longitudinal and clinical studies are recommended to explore causal relationships and guide evidence‐based interventions for LBP prevention and management.

### 6.1 Implications on Physiotherapy Practice

In Bangladesh, LBP is a common issue that affects people of all ages, including the younger generation. Policymakers should encourage early physiotherapy interventions and target high‐risk groups to reduce its prevalence. Reducing disability and long‐term complications requires early diagnosis and cause identification. LBP can be avoided by increasing public awareness through education and training initiatives, and those who are at risk should take preventative action. Effective LBP management also requires bolstering healthcare services, guaranteeing access to diagnostic centers, and encouraging regular exercise under medical supervision.

## Author Contributions

**Mohammad Omar Faruk:** conceptualization, data curation, formal analysis, investigation, methodology, project administration, resources, software, supervision, visualization, writing – original draft, writing – review and editing. **Najma Begum:** conceptualization, data curation, formal analysis, funding acquisition, methodology, resources, software, supervision, validation, visualization, writing – review and editing. **Kabir Hossain:** investigation, methodology, project administration, software, validation, visualization, writing – original draft, writing – review and editing. **Md. Rakib Rahman:** data curation, writing – review and editing. **Md. Sahidur Rahman:** supervision, writing – review and editing. **Sorif Hossain:** formal analysis, methodology, software, supervision, validation, writing – original draft, writing – review and editing.

## Ethics Statement

Ethical approval for this study was obtained from the Ethical Review Committee of the Noakhali Science and Technology University, Noakhali Science and Technology University, Noakhali‐3814, Bangladesh with Ref. No. NSTU/SCI/EC/2023/126.

## Consent

The authors obtained consent from the authority of the selected Center for the Rehabilitation of the Paralyzed to carry out this survey and obtained informed consent from each participant before conducting the interviews.

## Conflicts of Interest

The authors declare no conflicts of interest.

## Transparency Statement

The lead author Mohammad Omar Faruk affirms that this manuscript is an honest, accurate, and transparent account of the study being reported; that no important aspects of the study have been omitted; and that any discrepancies from the study as planned (and, if relevant, registered) have been explained.

## Supporting information

Supplementary.

## Data Availability

Research data supporting this publication will be available upon request from the corresponding author.
