# Supplementary material for: Risk Factors Associated With Low Back Pain in Bangladesh: A Cross‐Sectional Study Conducted in 2023
Source: Health Sci Rep. 2025 Aug 10;8(8):e71151. doi: 10.1002/hsr2.71151 (PMC12336289; doi:10.1002/hsr2.71151)
Supplement: Supplementary file 1 — Supplementary. [file HSR2-8-e71151-s001.docx]

**Table 5.** Cross-tabulation and Chi-square test of association between low back pain and

different socio-demographic, life style related and work-related factors in Bangladesh

|  |  | Grade I | Grade II | Grade III | Grade IV | P-value |
| --- | --- | --- | --- | --- | --- | --- |
| Gender | Female | 43 (10.9%) | 40(10.1%) | 101(25.5%) | 34(8.6%) | 0.158 |
|  | Male | 42(10.6%) | 45(11.4%) | 65(16.4%) | 26(6.6%) |  |
| Age of the participants | 18-30 | 35(8.8%) | 20(5.1%) | 23(5.8%) | 11(2.8%) | 0.000* |
|  | 31-40 | 19(4.8%) | 21(5.3%) | 35(8.8%) | 13(3.3%) |  |
|  | 41-50 | 14(3.5%) | 24(6.1%) | 48(12.1%) | 9(2.3%) |  |
|  | 51-60 | 12(3.0%) | 13(3.3%) | 41(10.4%) | 12(3.0%) |  |
|  | 60+ | 5(1.3%) | 7(1.8%) | 19(4.8%) | 15(3.8%) |  |
| Place of Residence | Rural | 21(5.3%) | 17(4.3%) | 63(15.9%) | 25(6.3%) | 0.005* |
|  | Urban | 64(16.2%) | 68(17.2%) | 103(26.0%) | 35(8.8%) |  |
| Education | No formal education | 5(1.3%) | 5(1.3%) | 28(7.1%) | 11(2.8%) | 0.000* |
|  | Primary | 8(2.0%) | 9(2.3%) | 32(8.1%) | 15(3.8%) |  |
|  | Secondary | 22(5.6%) | 15(3.8%) | 37(9.3%) | 14(3.5%) |  |
|  | Higher Secondary | 25(6.3%) | 22(5.6%) | 22(5.6%) | 7(1.8%) |  |
|  | Graduate | 21(5.3%) | 24(6.1%) | 29(7.3%) | 7(1.8%) |  |
|  | Post-graduate | 4(1.0%) | 10(2.5%) | 18(4.5%) | 6(1.5%) |  |
| Marital status | Married | 54(13.6%) | 63(15.9%) | 134(33.8%) | 44(11.1%) | 0.000* |
|  | Unmarried | 27(6.8%) | 15(3.8%) | 15(3.8%) | 6(1.5%) |  |
|  | Divorced/Widow/Separated | 4(1.0%) | 7(1.8%) | 17(4.3%) | 10(2.5%) |  |
| Income | 0 | 20(5.1%) | 18(4.5%) | 60(15.2%) | 19(4.8%) | 0.005* |
|  | 1-10 | 9(2.3%) | 7(1.8%) | 15(3.8%) | 12(3.0%) |  |
|  | 11-25 | 23(5.8%) | 13(3.3%) | 30(7.6%) | 15(3.8%) |  |
|  | 26-40 | 23(5.8%) | 24(6.1%) | 35(8.8%) | 8(2.0%) |  |
|  | 40+ | 10(2.5%) | 23(5.8%) | 26(6.6%) | 6(1.5%) |  |
| Occupation | Service Holder | 27(6.8%) | 25(6.3%) | 33(8.3%) | 15(3.8%) | 0.000* |
|  | Housewife | 14(3.5%) | 19(4.8%) | 73(18.4%) | 21(5.3%) |  |
|  | Teacher | 9(2.3%) | 10(2.5%) | 10(2.5%) | 1(0.3%) |  |
|  | Business | 3(0.8%) | 10(2.5%) | 16(4.0%) | 4(1.0%) |  |
|  | Student | 18(4.5%) | 8(2.0%) | 9(2.3%) | 6(1.5%) |  |
|  | Others | 14(3.5%) | 13(3.3%) | 25(6.3%) | 13(3.3%) |  |
| Family History (Parents) of LBP | No | 45(11.4%) | 56(14.1%) | 114(28.8%) | 41(10.4%) | 0.083 |
|  | Yes | 40(10.1%) | 29(7.3%) | 52(13.1%) | 19(4.8%) |  |
| Family History (Siblings) of LBP | No | 69(17.4%) | 71(17.9%) | 139(35.1%) | 51(12.9%) | 0.935 |
|  | Yes | 16(4.0%) | 14(3.5%) | 27(6.8%) | 9(2.3%) |  |
| Family History (Others) of LBP | No | 83(21.0%) | 82(20.7%) | 147(37.1%) | 54(13.6%) | 0.025* |
|  | Yes | 2(0.5%) | 3(0.8%) | 19(4.8%) | 6(1.5%) |  |
| Had other relevant illness (blood pressure) | No | 62(15.7%) | 57(14.4%) | 101(25.5%) | 36(9.1%) | 0.222 |
|  | Yes | 23(5.8%) | 28(7.1%) | 65(16.4%) | 24(6.1%) |  |
| Had other relevant illness (gastritis/ulcer) | No | 68(17.2%) | 65(16.4%) | 103(26.0%) | 34(8.6%) | 0.002* |
|  | Yes | 17(4.3%) | 20(5.1%) | 63(15.9%) | 26(6.6%) |  |
| Had other relevant illness (diabetes) | No | 75(18.9%) | 63(15.9%) | 112(28.3%) | 45(11.4%) | 0.005* |
|  | Yes | 10(2.5%) | 22(5.6%) | 54(13.6%) | 15(3.8%) |  |
| Had other relevant illness (cough) | No | 81(20.5%) | 73(18.4%) | 145(36.6%) | 45(11.4%) | 0.005* |
|  | Yes | 4(1.0%) | 12(3.0%) | 21(5.3%) | 15(3.8%) |  |
| Had other's problem | No | 78(19.7%) | 80(20.2%) | 154(38.9%) | 52(13.1%) | 0.400 |
|  | Yes | 7(1.8%) | 5(1.3%) | 12(3.0%) | 8(2.0%) |  |
| Keeping a wallet or something in the pocket | Back Pocket | 26(6.6%) | 34(8.6%) | 37(9.3%) | 11(2.8%) | 0.026* |
|  | Front Pocket | 6(1.5%) | 6(1.5%) | 14(3.5%) | 9(2.3%) |  |
|  | None | 53(13.4%) | 45(11.4%) | 115(29.0%) | 40(10.1%) |  |
| Have you ever had an incidence of discomfort seating or trauma? | Yes | 20(5.1%) | 33(8.3%) | 82(20.7%) | 44(11.1%) | 0.000* |
|  | No | 56(14.1%) | 41(10.4%) | 58(14.6%) | 9(2.3%) |  |
|  | Don't Know | 9(2.3%) | 11(2.8%) | 26(6.6%) | 7(1.8%) |  |
| Type of sleeping bed | Soft | 12(3.0%) | 22(5.6%) | 38(9.6%) | 21(5.3%) | 0.167 |
|  | Moderate | 60(15.2%) | 51(12.9%) | 106(26.8%) | 31(7.8%) |  |
|  | Hard | 13(3.3%) | 12(3.0%) | 22(5.6%) | 8(2.0%) |  |
| Pillow height | Low | 14(3.5%) | 13(3.3%) | 42(10.6%) | 17(4.3%) | 0.021* |
|  | Moderate | 67(16.9%) | 65(16.4%) | 102(25.8%) | 34(8.6%) |  |
|  | High | 4(1.0%) | 7(1.8%) | 22(5.6%) | 9(2.3%) |  |
| Type of pillow | Soft | 18(4.5%) | 20(5.1%) | 64(16.2%) | 28(7.1%) | 0.004* |
|  | Moderate | 61(15.4%) | 62(15.7%) | 90(22.7%) | 30(7.6%) |  |
|  | Hard | 6(1.5%) | 3(0.8%) | 12(3.0%) | 2(0.5%) |  |
| Sleeping position | Left/Right | 53(13.4%) | 63(15.9%) | 116(29.3%) | 35(8.8%) | 0.292 |
|  | Straight | 26(6.6%) | 18(4.5%) | 42(10.6%) | 18(4.5%) |  |
|  | Back | 6(1.5%) | 4(1.0%) | 8(2.0%) | 7(1.8%) |  |
| Smoking habit | None | 54(13.6%) | 57(14.4%) | 131(33.1%) | 47(11.9%) | 0.066 |
|  | Occasionally | 8(2.0%) | 8(2.0%) | 5(1.3%) | 0(0.0%) |  |
|  | Frequent | 6(1.5%) | 5(1.3%) | 10(2.5%) | 5(1.3%) |  |
|  | Regular | 17(4.3%) | 15(3.8%) | 20(5.1%) | 8(2.0%) |  |
| Physical exercise | None | 35(8.8%) | 40(10.1%) | 56(14.1%) | 15(3.8%) | 0.107 |
|  | Occasionally | 14(3.5%) | 15(3.8%) | 27(6.8%) | 8(2.0%) |  |
|  | Frequent | 14(3.5%) | 15(3.8%) | 28(7.1%) | 12(3.0%) |  |
|  | Regular | 22(5.6%) | 15(3.8%) | 55(13.9%) | 25(6.3%) |  |
| Commode type used in bathroom | Low | 60(15.2%) | 56(14.1%) | 99(25.0%) | 35(8.8%) | 0.238 |
|  | High | 24(6.1%) | 29(7.3%) | 66(16.7%) | 23(5.8%) |  |
|  | Others | 1(0.3%) | 0(0.0%) | 1(0.3%) | 2(0.5%) |  |
| Strenuous physical activity | Not at all | 60(15.2%) | 46(11.6%) | 58(14.6%) | 18(4.5%) | 0.000* |
|  | Rarely | 10(2.5%) | 24(6.1%) | 43(10.9%) | 25(6.3%) |  |
|  | Sometimes | 14(3.5%) | 14(3.5%) | 56(14.1%) | 12(3.0%) |  |
|  | Regular | 1(0.3%) | 1(0.3%) | 9(2.3%) | 5(1.3%) |  |
| Leisure time activity (watching TV/mobile/reading books) | No | 21(5.3%) | 19(4.8%) | 28(7.1%) | 12(3.0%) | 0.483 |
|  | Yes | 64(16.2%) | 66(16.7%) | 138(34.8%) | 48(12.1%) |  |
| Leisure time activity (hanging with friends) | No | 63(15.9%) | 60(15.2%) | 139(35.1%) | 52(13.1%) | 0.026* |
|  | Yes | 22(5.6%) | 25(6.3%) | 27(6.8%) | 8(2.0%) |  |
| Leisure time activity (moving activities) | No | 80(20.2%) | 81(20.5%) | 159(40.2%) | 57(14.4%) | 0.951 |
|  | Yes | 5(1.3%) | 4(1.0%) | 7(1.8%) | 3(0.8%) |  |
| Average Sleeping Time | 4.00 | 2(0.5%) | 6(1.5%) | 18(4.5%) | 6(1.5%) | 0.294 |
|  | 5.00 | 6(1.5%) | 13(3.3%) | 13(3.3%) | 6(1.5%) |  |
|  | 6.00 | 30(7.6%) | 32(8.1%) | 53(13.4%) | 16(4.0%) |  |
|  | 6.50 | 1(0.3%) | 0(0.0%) | 2(0.5%) | 0(0.0%) |  |
|  | 7.00 | 24(6.1%) | 16(4.0%) | 41(10.4%) | 12(3.0%) |  |
|  | 8.00 | 18(4.5%) | 11(2.8%) | 29(7.3%) | 12(3.0%) |  |
|  | 9.00 | 4(1.0%) | 7(1.8%) | 10(2.5%) | 8(2.0%) |  |
| Time spent in transport (in hours) | 0-1 | 76(19.2%) | 68(17.2%) | 135(34.1%) | 50(12.6%) | 0.598 |
|  | 1-2 | 6(1.5%) | 7(1.8%) | 15(3.8%) | 5(1.3%) |  |
|  | 2+ | 3(0.8%) | 10(2.5%) | 16(4.0%) | 5(1.3%) |  |
| Do you feel stress in your job? | Never | 37(9.3%) | 18(4.5%) | 27(6.8%) | 9(2.3%) | 0.000* |
|  | Rarely | 20(5.1%) | 26(6.6%) | 44(11.1%) | 19(4.8%) |  |
|  | Frequently | 22(5.6%) | 28(7.1%) | 65(16.4%) | 17(4.3%) |  |
|  | Always | 6(1.5%) | 13(3.3%) | 30(7.6%) | 15(3.8%) |  |
| What type of chair do you use in your working area? | Plastic Chair | 14(3.5%) | 13(3.3%) | 49(12.4%) | 20(5.1%) | 0.002* |
|  | Soft leather/cushion Chair | 18(4.5%) | 30(7.6%) | 38(9.6%) | 7(1.8%) |  |
|  | Wooden Chair | 29(7.3%) | 27(6.8%) | 37(9.3%) | 13(3.3%) |  |
|  | No use of chair | 24(6.1%) | 15(3.8%) | 42(10.6%) | 20(5.1%) |  |
| Nature of work of your job | Seating | 35(8.8%) | 36(9.1%) | 47(11.9%) | 15(3.8%) | 0.008* |
|  | Standing | 9(2.3%) | 6(1.5%) | 29(7.3%) | 16(4.0%) |  |
|  | Frequent Movement | 41(10.4%) | 43(10.9%) | 90(22.7%) | 29(7.3%) |  |
| Weight of shoe in last 6 months | Light | 34(8.6%) | 55(13.9%) | 55(13.9%) | 27(6.8%) | 0.000* |
|  | Medium | 49(12.4%) | 29(7.3%) | 95(24.0%) | 26(6.6%) |  |
|  | Heavy | 2(0.5%) | 1(0.3%) | 16(4.0%) | 7(1.8%) |  |
| Do you drive/use the bike? | None | 57(14.4%) | 60(15.2%) | 132(33.3%) | 45(11.4%) | 0.149 |
|  | Occasionally | 7(1.8%) | 11(2.8%) | 13(3.3%) | 4(1.0%) |  |
|  | Frequent | 12(3.0%) | 4(1.0%) | 7(1.8%) | 6(1.5%) |  |
|  | Regular | 9(2.3%) | 10(2.5%) | 14(3.5%) | 5(1.3%) |  |
| Do you work overtime? | Never | 56(14.1%) | 38(9.6%) | 87(22.0%) | 30(7.6%) | 0.163 |
|  | Sometimes | 24(6.1%) | 39(9.8%) | 68(17.2%) | 27(6.8%) |  |
|  | Regularly | 5(1.3%) | 8(2.0%) | 11(2.8%) | 3(0.8%) |  |
| Total number of years actively worked (years) | 0-10 | 54(13.6%) | 46(11.6%) | 99(25.0%) | 49(12.4%) | 0.161 |
|  | 10-20 | 15(3.8%) | 20(5.1%) | 28(7.1%) | 4(1.0%) |  |
|  | 20-30 | 11(2.8%) | 15(3.8%) | 27(6.8%) | 5(1.3%) |  |
|  | 30-40 | 5(1.3%) | 3(0.8%) | 9(2.3%) | 2(0.5%) |  |
|  | 40+ | 0(0.0%) | 1(0.3%) | 3(0.8%) | 0(0.0%) |  |
| Daily seating hours | 0-4 | 30(7.6%) | 31(7.8%) | 89(22.5%) | 27(6.8%) | 0.049* |
|  | 4-8 | 45(11.4%) | 42(10.6%) | 67(16.9%) | 27(6.8%) |  |
|  | 8-12 | 10(2.5%) | 12(3.0%) | 10(2.5%) | 6(1.5%) |  |
| WORKING Hours | 1-4 | 8(2.0%) | 12(3.0%) | 31(7.8%) | 15(3.8%) | 0.170 |
|  | 5-8 | 61(15.4%) | 54(13.6%) | 108(27.3%) | 38(9.6%) |  |
|  | 9-12 | 16(4.0%) | 19(4.8%) | 27(6.8%) | 7(1.8%) |  |
| Daily Standing Hours | 0-3 | 48(12.1%) | 55(13.9%) | 113(28.5%) | 35(8.8%) | 0.511 |
|  | 3-6 | 28(7.1%) | 22(5.6%) | 38(9.6%) | 16(4.0%) |  |
|  | 6-9 | 9(2.3%) | 8(2.0%) | 15(3.8%) | 9(2.3%) |  |
| Daily Walking Hours | 0-1 | 40(10.1%) | 58(14.6%) | 103(26.0%) | 35(8.8%) | 0.139 |
|  | 1-2 | 25(6.3%) | 15(3.8%) | 33(8.3%) | 11(2.8%) |  |
|  | 3 | 20(5.1%) | 12(3.0%) | 30(7.6%) | 14(3.5%) |  |
| Note: * significant at 5% level | | | | | | |

**Table 6.** Multinomial logistic regression analysis to assess factors associated with LBP

|  | **Grade II** | | |  | **Grade III** | | |  | **Grade IV** | | |  |
| --- | --- | --- | --- | --- | --- | --- | --- | --- | --- | --- | --- | --- |
| **Variables** | **Exp(B)** | **95% CI for Exp(B)** | | **Sig.** | **Exp(B)** | **95% CI for Exp(B)** | | **Sig.** | **Exp(B)** | **95% CI for Exp(B)** | | **Sig.** |
|  |  | **Lower Bound** | **Upper Bound** |  |  | **Lower Bound** | **Upper Bound** |  |  | **Lower Bound** | **Upper Bound** |  |
| **Age of the participants** | | | | | | | | | | | | |
| 18-30 | 0.504 | 0.046 | 5.574 | 0.576 | 0.147 | 0.016 | 1.353 | 0.090 | 0.040 | 0.002 | 0.724 | 0.029* |
| 31-40 | 0.265 | 0.032 | 2.174 | 0.216 | 0.291 | 0.043 | 1.994 | 0.209 | 0.055 | 0.005 | 0.647 | 0.021* |
| 41-50 | 0.503 | 0.063 | 4.051 | 0.519 | 0.452 | 0.066 | 3.101 | 0.419 | 0.030 | 0.002 | 0.353 | 0.005* |
| 51-60 | 0.164 | 0.020 | 1.378 | 0.096 | 0.745 | 0.108 | 5.131 | 0.765 | 0.176 | 0.016 | 1.899 | 0.152 |
| 60+ | Reference |  |  |  |  |  |  |  |  |  |  |  |
| **Place of Residence** | | | | | | | | | | | |  |
| Rural | 0.617 | 0.197 | 1.933 | 0.407 | 2.659 | 0.973 | 7.266 | 0.057 | 3.432 | 0.955 | 12.333 | 0.059 |
| Urban | Reference |  |  |  |  |  |  |  |  |  |  |  |
| **Education** | | | | | | | | | | | | |
| No formal education | 0.376 | 0.020 | 6.935 | 0.510 | 0.178 | 0.013 | 2.393 | 0.193 | 0.106 | 0.004 | 2.722 | 0.175 |
| Primary | 1.056 | 0.068 | 16.419 | 0.969 | 0.562 | 0.045 | 7.091 | 0.656 | 0.360 | 0.016 | 8.114 | 0.520 |
| Secondary | 0.404 | 0.040 | 4.037 | 0.440 | 0.098 | 0.011 | 0.831 | 0.033* | 0.080 | 0.005 | 1.202 | 0.068 |
| Higher Secondary | 0.791 | 0.093 | 6.691 | 0.829 | 0.123 | 0.017 | 0.916 | 0.041* | 0.071 | 0.005 | 0.987 | 0.049* |
| Graduate | 2.181 | 0.260 | 18.323 | 0.473 | 0.442 | 0.063 | 3.098 | 0.411 | 0.174 | 0.012 | 2.534 | 0.201 |
| Post-graduate | Reference |  |  |  |  |  |  |  |  |  |  |  |
| **Marital Status** | | | | | | | | | | | | |
| Married | 0.278 | 0.041 | 1.904 | 0.192 | 0.768 | 0.130 | 4.555 | 0.772 | 0.297 | 0.033 | 2.699 | 0.281 |
| Unmarried | 0.175 | 0.013 | 2.441 | 0.195 | 0.871 | 0.068 | 11.153 | 0.915 | 0.016 | 0.000 | 0.796 | 0.038* |
| Divorced | Reference |  |  |  |  |  |  |  |  |  |  |  |
| **Income** | | | | | | | | | | | | |
| 0 | 2.622 | 0.312 | 22.007 | 0.375 | 1.198 | 0.147 | 9.752 | 0.866 | 2.284 | 0.151 | 34.488 | 0.551 |
| 1--10 | 0.650 | 0.063 | 6.673 | 0.717 | 0.896 | 0.105 | 7.676 | 0.920 | 8.804 | 0.487 | 159.087 | 0.141 |
| 11--25 | 0.286 | 0.048 | 1.718 | 0.171 | 0.681 | 0.122 | 3.801 | 0.661 | 1.471 | 0.131 | 16.535 | 0.755 |
| 26--40 | 0.303 | 0.068 | 1.356 | 0.118 | 0.291 | 0.066 | 1.282 | 0.103 | 0.778 | 0.076 | 7.976 | 0.833 |
| 40+ | Reference |  |  |  |  |  |  |  |  |  |  |  |
| **Occupation** | | | | | | | | | | | | |
| Service Holder | 0.557 | 0.089 | 3.479 | 0.532 | 0.472 | 0.095 | 2.350 | 0.359 | 0.830 | 0.106 | 6.483 | 0.859 |
| Housewife | 1.281 | 0.130 | 12.629 | 0.832 | 2.010 | 0.263 | 15.343 | 0.501 | 2.713 | 0.229 | 32.101 | 0.429 |
| Teacher | 1.676 | 0.180 | 15.650 | 0.650 | 0.859 | 0.098 | 7.506 | 0.891 | 0.219 | 0.005 | 9.837 | 0.434 |
| Business | 4.442 | 0.438 | 45.004 | 0.207 | 4.119 | 0.484 | 35.084 | 0.195 | 6.722 | 0.408 | 110.786 | 0.183 |
| Student | 0.061 | 0.004 | 0.881 | 0.040* | 0.179 | 0.013 | 2.434 | 0.196 | 10.655 | 0.177 | 640.609 | 0.258 |
| Others | Reference |  |  |  |  |  |  |  |  |  |  |  |
| **Family History (Others) of LBP** | | | | | | | | | | | | |
| No | 1.826 | 0.117 | 28.410 | 0.667 | 0.216 | 0.021 | 2.215 | 0.197 | 0.201 | 0.012 | 3.411 | 0.266 |
| Yes | Reference |  |  |  |  |  |  |  |  |  |  |  |
| **Had other relevant illness (gastritis/ulcer)** | | | | | | | | | | | | |
| No | 1.166 | 0.367 | 3.707 | 0.794 | 0.570 | 0.193 | 1.685 | 0.309 | 0.465 | 0.119 | 1.824 | 0.272 |
| Yes | Reference |  |  |  |  |  |  |  |  |  |  |  |
| **Had other relevant illness (diabetes)** | | | | | | | | | | | | |
| No | 0.262 | 0.073 | 0.939 | 0.040* | 0.284 | 0.087 | 0.931 | 0.038* | 0.758 | 0.154 | 3.739 | 0.734 |
| Yes | Reference |  |  |  |  |  |  |  |  |  |  |  |
| **Had other relevant illness (cough)** | | | | | | | | | | | | |
| No | 0.193 | 0.028 | 1.330 | 0.095 | 0.095 | 0.016 | 0.563 | 0.009 | 0.142 | 0.019 | 1.046 | 0.055 |
| Yes | Reference |  |  |  |  |  |  |  |  |  |  |  |
| **Keeping a wallet or something in the pocket** | | | | | | | | | | | | |
| Back Pocket | 1.155 | 0.320 | 4.172 | 0.826 | 0.329 | 0.100 | 1.081 | 0.067 | 0.271 | 0.052 | 1.424 | 0.123 |
| Front Pocket | 1.416 | 0.211 | 9.492 | 0.720 | 0.822 | 0.146 | 4.630 | 0.824 | 2.313 | 0.261 | 20.457 | 0.451 |
| None | Reference |  |  |  |  |  |  |  |  |  |  |  |
| **Have you ever had an incidence of discomfort sitting or trauma?** | | | | | | | | | | | | |
| Yes | 1.603 | 0.356 | 7.224 | 0.539 | 1.096 | 0.273 | 4.396 | 0.897 | 1.871 | 0.312 | 11.222 | 0.493 |
| No | 1.384 | 0.318 | 6.030 | 0.665 | 0.408 | 0.106 | 1.570 | 0.192 | 0.130 | 0.019 | 0.888 | 0.037* |
| Don't Know | Reference |  |  |  |  |  |  |  |  |  |  |  |
| **Pillow height** | | | | | | | | | | | | |
| Low | 0.638 | 0.081 | 5.039 | 0.670 | 0.722 | 0.113 | 4.596 | 0.730 | 0.491 | 0.048 | 4.992 | 0.548 |
| Moderate | 0.455 | 0.070 | 2.966 | 0.410 | 0.355 | 0.062 | 2.036 | 0.245 | 0.347 | 0.041 | 2.951 | 0.332 |
| High | Reference |  |  |  |  |  |  |  |  |  |  |  |
| **Type of pillow** | | | | | | | | | | | | |
| Soft | 1.491 | 0.101 | 21.967 | 0.771 | 6.596 | 0.873 | 49.815 | 0.067 | 1.684 | 1.055 | 2.69 | 0.027* |
| Moderate | 1.201 | 0.092 | 15.677 | 0.889 | 3.014 | 0.438 | 20.761 | 0.263 | 1.212 | 1.014 | 1.806 | 0.048* |
| Hard | Reference |  |  |  |  |  |  |  |  |  |  |  |
| **Strenuous physical activity** | | | | | | | | | | | | |
| Not at all | 4.797 | 0.133 | 172.667 | 0.391 | 0.691 | 0.041 | 11.762 | 0.798 | 0.290 | 0.012 | 7.293 | 0.452 |
| Rarely | 51.018 | 1.258 | 2068.829 | 0.037* | 4.375 | 0.217 | 88.080 | 0.335 | 3.541 | 0.123 | 102.201 | 0.461 |
| Sometimes | 15.331 | 0.404 | 581.856 | 0.141 | 2.973 | 0.169 | 52.411 | 0.457 | 0.812 | 0.032 | 20.708 | 0.900 |
| Regular | Reference |  |  |  |  |  |  |  |  |  |  |  |
| **Leisure time activity (hanging with friends)** | | | | | | | | | | | | |
| No | 0.272 | 0.083 | 0.890 | 0.031* | 0.434 | 0.136 | 1.387 | 0.159 | 1.489 | 0.283 | 7.825 | 0.638 |
| Yes | Reference |  |  |  |  |  |  |  |  |  |  |  |
| **Do you feel stress in your job?** | | | | | | | | | | | |  |
| Never | 0.072 | 0.011 | 0.466 | 0.006* | 0.268 | 0.047 | 1.525 | 0.138 | 0.197 | 0.022 | 1.724 | 0.142 |
| Rarely | 0.260 | 0.043 | 1.592 | 0.145 | 1.277 | 0.230 | 7.101 | 0.780 | 0.553 | 0.072 | 4.246 | 0.569 |
| Frequently | 0.251 | 0.044 | 1.418 | 0.118 | 1.042 | 0.218 | 4.972 | 0.959 | 0.332 | 0.050 | 2.200 | 0.253 |
| Always | Reference |  |  |  |  |  |  |  |  |  |  |  |
| **What type of chair do you use in your working area?** | | | | | | | | | | | | |
| Plastic Chair | 1.636 | 0.362 | 7.391 | 0.522 | 2.582 | 0.687 | 9.712 | 0.160 | 1.169 | 0.211 | 6.472 | 0.858 |
| Soft leather/cushion Chair | 6.174 | 1.185 | 32.178 | 0.031* | 2.667 | 0.544 | 13.091 | 0.227 | 0.206 | 0.023 | 1.829 | 0.156 |
| Wooden Chair | 2.279 | 0.602 | 8.627 | 0.225 | 1.880 | 0.537 | 6.577 | 0.323 | 0.794 | 0.142 | 4.453 | 0.793 |
| No use of chair | Reference |  |  |  |  |  |  |  |  |  |  |  |
| **Nature of work of your job** | | | | | | | | | | | | |
| Seating | 0.462 | 0.151 | 1.413 | 0.176 | 0.797 | 0.284 | 2.235 | 0.666 | 0.925 | 0.211 | 4.048 | 0.917 |
| Standing | 0.398 | 0.071 | 2.221 | 0.293 | 1.642 | 0.401 | 6.723 | 0.491 | 7.206 | 1.304 | 39.828 | 0.024* |
| Frequent Movement | Reference |  |  |  |  |  |  |  |  |  |  |  |
| **Weight of shoe in last 6 months** | | | | | | | | | | | | |
| Light | 1.069 | 0.033 | 34.397 | 0.970 | 0.020 | 0.001 | 0.336 | 0.007* | 0.027 | 0.001 | 0.677 | 0.028* |
| Medium | 0.447 | 0.014 | 14.103 | 0.648 | 0.034 | 0.002 | 0.570 | 0.019* | 0.027 | 0.001 | 0.667 | 0.027* |
| Heavy | Reference |  |  |  |  |  |  |  |  |  |  |  |
| **Daily seating hours** | | | | | | | | | | | | |
| 0-4 | 0.581 | 0.094 | 3.579 | 0.558 | 2.755 | 0.457 | 16.624 | 0.269 | 1.611 | 0.151 | 17.239 | 0.693 |
| 4--8 | 0.256 | 0.053 | 1.232 | 0.089 | 1.342 | 0.267 | 6.754 | 0.721 | 1.367 | 0.153 | 12.196 | 0.780 |
| 8--12 | Reference |  |  |  |  |  |  |  |  |  |  |  |
